# Supplementary material for: Risk of Dry Eye Syndrome in Patients with Orbital Fracture: A Nationwide Population-Based Cohort Study
Source: Healthcare (Basel). 2021 May 18;9(5):605. doi: 10.3390/healthcare9050605 (PMC8157863; doi:10.3390/healthcare9050605)
Supplement: Supplementary file 1 [file healthcare-09-00605-s001.zip › healthcare-1198963-supplementary.pdf]

Supplementary

Table S1. Years of follow-up

Orbital fracture	Min	Median	Max	Mean ± SD
With	0.01	6.97	15.95	10.29 ± 15.95
Without	0.01	8.79	15.99	10.75 ± 9.91
Total	0.01	7.15	15.99	10.66 ± 11.38

Table S2. Years to dry eye syndrome

Orbital fracture	Min	Median	Max	Mean ± SD
With	0.01	3.72	11.94	4.13 ± 2.84
Without	0.02	6.56	15.76	6.91 ± 4.57
Total	0.01	5.50	15.76	6.14 ± 4.34

Table S3. The incidence of DES in the orbital fracture group and in the general population group

**Orbital fracture In the tracking of x year(s)**	**With** (n = 46,179)	**Without** (n = 184,716)	**Log-rank *p***
	**Numbers of dry eye syndrome**		
1	14	26	0.598
2	19	39	0.444
3	28	52	0.036
4	43	64	<0.001
5	52	84	<0.001
6	60	99	<0.001
7	67	110	<0.001
8	72	123	<0.001
9	74	135	<0.001
10	75	145	<0.001
11	78	155	<0.001
12	79	167	<0.001
13	79	183	<0.001
14	79	192	<0.001
15	79	199	<0.001
16	79	206	<0.001
